# Detection of Motor Dysfunction With Wearable Sensors in Patients With Idiopathic Rapid Eye Movement Disorder

**DOI:** 10.3389/fbioe.2021.627481

**Published:** 2021-04-15

**Authors:** Lin Ma, Shu-Ying Liu, Shan-Shan Cen, Yuan Li, Hui Zhang, Chao Han, Zhu-Qin Gu, Wei Mao, Jing-Hong Ma, Yong-Tao Zhou, Er-He Xu, Piu Chan

**Affiliations:** ^1^Department of Neurobiology, Neurology and Geriatrics, Xuanwu Hospital of Capital Medical University, Beijing Institute of Geriatrics, Beijing, China; ^2^National Clinical Research Center for Geriatric Disorders, Beijing, China; ^3^Clinical and Research Center for Parkinson’s Disease, Capital Medical University, Beijing, China; ^4^Key Laboratory for Neurodegenerative Disease of the Ministry of Education, Beijing Key Laboratory for Parkinson’s Disease, Parkinson Disease Center of Beijing Institute for Brain Disorders, Beijing, China; ^5^Advanced Innovation Center for Human Brain Protection, Capital Medical University, Beijing, China

**Keywords:** Idiopathic REM sleep behavior disorder, gait, quantitative measurement, prodromal stage, wearable sensors

## Abstract

Patients with idiopathic rapid eye movement sleep behavior disorder (iRBD) are at high risk for conversion to synucleinopathy and Parkinson disease (PD). This can potentially be monitored by measuring gait characteristics of iRBD patients, although quantitative data are scarce and previous studies have reported inconsistent findings. This study investigated subclinical gait changes in polysomnography-proven iRBD patients compared to healthy controls (HCs) during 3 different walking conditions using wearable motor sensors in order to determine whether gait changes can be detected in iRBD patients that could reflect early symptoms of movement disorder. A total 31 iRBD patients and 20 HCs were asked to walk in a 10-m corridor at their usual pace, their fastest pace, and a normal pace while performing an arithmetic operation (dual-task condition) for 1 min each while using a wearable gait analysis system. General gait measurements including stride length, stride velocity, stride time, gait length asymmetry, and gait variability did not differ between iRBD patients and HCs; however, the patients showed decreases in range of motion (*P* = 0.004) and peak angular velocity of the trunk (*P* = 0.001) that were significant in all 3 walking conditions. iRBD patients also had a longer step time before turning compared to HCs (*P* = 0.035), and the difference between groups remained significant after adjusting for age, sex, and height. The decreased trunk motion while walking and increased step time before turning observed in iRBD may be early manifestations of body rigidity and freezing of gait and are possible prodromal symptoms of PD.

## Introduction

Rapid eye movement (REM) sleep behavior disorder (RBD) is characterized by episodes of vigorous movements during REM sleep, usually accompanied by unpleasant dreams and violent limb movements (Iranzo et al., 2016). Up to 97% of patients with idiopathic (i) RBD progress within 14.2 years to synucleinopathies such as Parkinson disease (PD), multiple system atrophy, and dementia with Lewy bodies (Galbiati et al., 2019). According to the Movement Disorder Society Research Criteria for Prodromal Parkinson’s Disease, polysomnography (PSG)-proven RBD is the most significant risk factor for prodromal PD (Heinzel et al., 2019). Thus, patients diagnosed with iRBD are potential candidates for clinical trials of neuroprotective therapies (Postuma et al., 2015).

Wearable sensors can provide reliable and unbiased data on subtle changes in gait. Previous studies have used sensors to objectively analyze gait abnormality in PD (Silva de Lima et al., 2017; Suzuki et al., 2017) and other types of parkinsonism (Raccagni et al., 2018). These studies demonstrated that quantitative gait characteristics can be used to identify prodromal PD, and that higher gait variability and asymmetry during a single task at the usual walking speed can predict time to PD conversion (Del Din et al., 2019).

There have been few studies of quantitative motor assessment in RBD patients, and the results are inconsistent. One study found that probable RBD diagnosed with the Mayo Sleep Questionnaire was associated with decreased velocity and cadence and increased stride time variability as measured using the GAITRite system (a 5.0 × 0.7-m pressure sensor walkway) (McDade et al., 2013); however, decreases in gait velocity, rhythm, and gait variability were observed by real-world gait monitoring of PSG-proven iRBD patients using a tri-axial accelerometer (Del Din et al., 2020). In another study in which a 6.1 m × 0.61 m Zeno pressure sensor walkway was used to measure gait, no differences in step length and velocity were observed between PSG-proven iRBD patients and healthy controls (HCs); however, during fast-paced walking, iRBD patients showed greater gait asymmetry and in the dual-task walking condition, step width variability was increased (Ehgoetz Martens et al., 2019). iRBD patients also showed impairment in biomechanical measures of self-initiated stepping including reductions in the posterior shift of the center of pressure during the anticipatory and propulsive phases of gait initiation that resembled the freezing of gait (FOG) observed in PD (Alibiglou et al., 2016).

Most studies have used pressure sensors to measure RBD patients’ gait. Wearable sensors are composed of a tri-axial accelerometer, gyroscope, and magnetometer and have the advantages of being small and lightweight with wireless transmission, which make the devices portable and convenient to use outside the laboratory and in long-term daily monitoring; moreover, the devices can be used to collect data on trunk and arm movements.

In this study, we used wearable motor sensors to detect subclinical gait changes and quantitatively analyze motor performance of PSG-confirmed iRBD patients compared to HC subjects. We also examined whether iRBD patients with greater gait abnormality were at a higher risk of conversion to synucleinopathy. Our results indicate that iRBD is associated with decreased trunk motion while walking and increased step time before turning, which may be prodromal symptoms of PD.

## Patients and Methods

### Participants

The iRBD patients were recruited from the neurology clinic of Xuanwu Hospital, Beijing, China (Li et al., 2019) and HCs were recruited from a community-based cohort study conducted in Beijing (Ji et al., 2020) over a 3-year period (2013–2015). iRBD patients were PSG-confirmed and had not been diagnosed with any neurodegenerative disease. Patients were excluded if they had a total score < 18 for the Rapid Eye Movement (REM) Sleep Behavior Disorder Questionnaire – Hong Kong (RBDQ-HK) (Li et al., 2010), obstructive sleep apnea-hypopnea syndrome or any other sleep disorder, musculoskeletal conditions, or prior surgeries that could influence gait. The study was approved by the Ethics Committee of Xuanwu Hospital Capital Medical and all participants provided written, informed consent.

### Procedures

Demographic data and medical history were recorded. All participants underwent a comprehensive neurologic assessment that included Part III of the Unified Parkinson’s Disease Rating Scale (UPDRS III) to assess motor symptoms, Montreal Cognitive Assessment (MoCA) to assess cognitive state, RBDQ-HK to screen for RBD, a 5-odor olfactory detection array to evaluate the threshold of olfactory identification (TOI) (Cao et al., 2016), and Non-motor Symptoms Scale (NMSS) to measure the number and severity of non-motor symptoms.

### Gait Assessment

**FIGURE 1:**
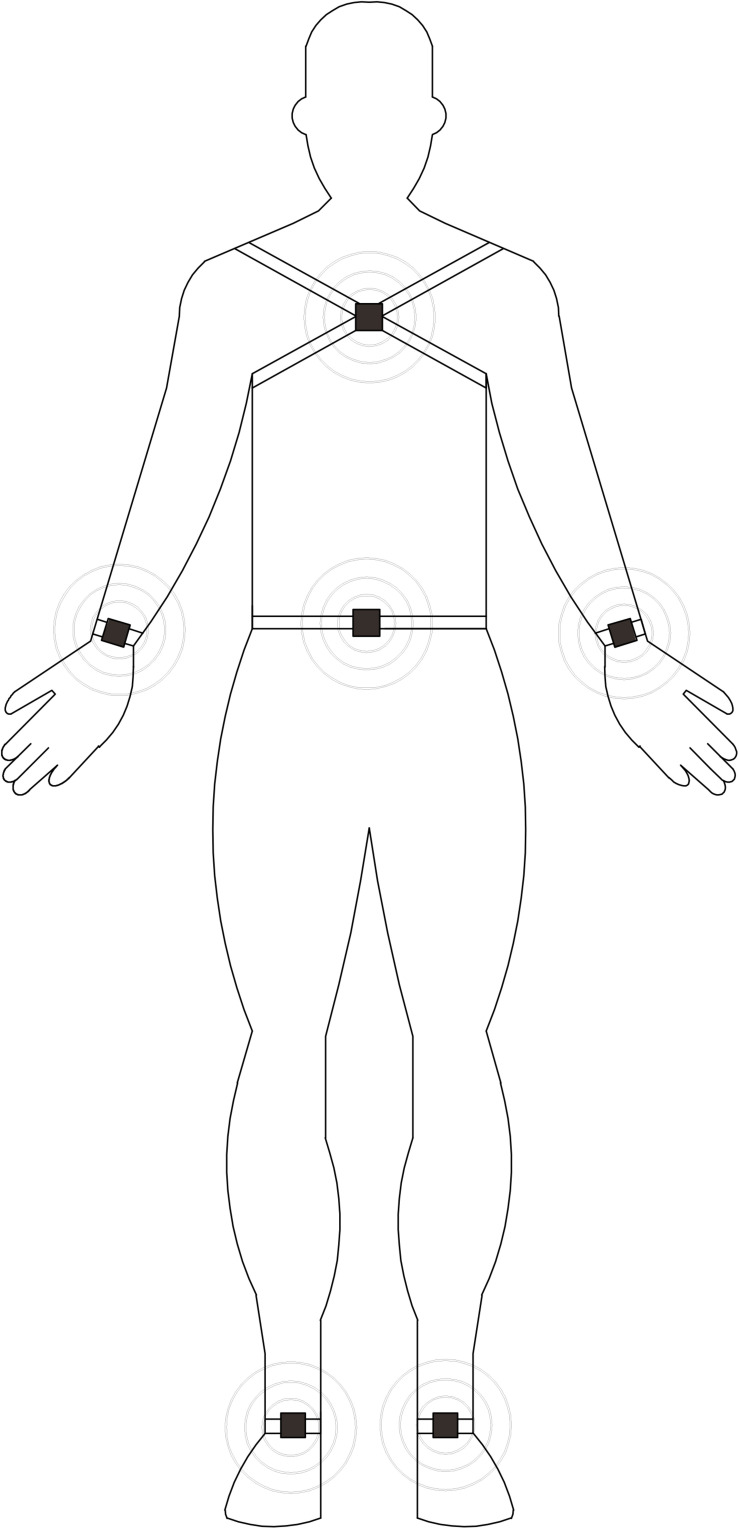
Location of sensors on the body.

Participants completed 3 walking trials with a wearable system for quantitative gait analysis comprising 6 wearable gyroscope and accelerometer sensors (APDM; Mobility Lab, Portland, OR, United States). The sensors were placed at bilateral wrists and ankles, the anterior sternum, and lower back (Figure 1). Participants were asked to walk in a corridor with a 10-m effective distance at their usual pace, fastest pace, and a normal pace while subtracting 7 from 100 (dual-task condition) for 1 min each (an average of 22 valid strides per walking condition). Participants were instructed to walk past a line marked with tape and turn during each task.

The following parameters were examined in the study based on previous reports (Salarian et al., 2010; Washabaugh et al., 2017).

• Normalized stride length, which is the distance between 2 consecutive footfalls at the moment of initial contact; the value is normalized for height and averaged for left and right sides.
• Normalized stride velocity, which is the participant’s walking speed normalized to his/her height and averaged for left and right sides.
• Stride time, which is the duration of a complete gait cycle (defined as the period between 2 consecutive initial contacts [heel strikes] of the right foot).
• Range of motion of the trunk in the sagittal plane, which is the angular range of the thoracic spine in the anterior–posterior plane (i.e., moving back and forth).
• Range of motion of the trunk in the horizontal plane, which is the angular range of the thoracic spine in the head–feet plane (i.e., moving up and down).
• Peak angular velocity of the trunk in the sagittal or horizontal plane, which is the peak angular speed of the thoracic spine motion in the sagittal or horizontal plane, respectively.
• Step time before turning, which is the duration of the last step immediately before a turn.
• Stride length asymmetry, which is the mean asymmetry of the left and right stride lengths and is calculated as $100\times \left|\ln\,\left\{\frac{\text{min}\,\left(s\,t\,r\,i\,d\,e\,l\,e\,n\,g\,t\,h\,L,s\,t\,r\,i\,d\,e\,l\,e\,n\,g\,t\,h\,R\right)}{\text{max}\,\left(s\,t\,r\,i\,d\,e\,l\,e\,n\,g\,t\,h\,L,s\,t\,r\,i\,d\,e\,l\,e\,n\,g\,t\,h\,R\right)}\right\}\right|$ (Sant’Anna et al., 2011).
• Coefficient of variation of stride length, which is the stride length variability of multiple consecutive strides and is calculated as 100 × standard deviation/mean value of stride length.
• Coefficient of variation of stride time, which is the stride time variability of multiple consecutive strides and is calculated as 100 × standard deviation/mean value of stride time.

### Statistical Analysis

All parameters were checked for normality and homoscedasticity within groups. Repeated-measures analysis of variance was used to assess between-group effects (iRBD vs. HC), the effect of the walking condition (usual, fast, or dual-task), and any interactions between group and walking condition. When group or walking condition effect differences were significant, posthoc analyses were performed with Bonferroni correction. The covariance analysis method was used to adjust for covariates; normalized stride length and velocity were adjusted for age and sex as they were already normalized by height; the other variables were adjusted for age, sex, and height. The independent-samples *t* test and Mann–Whitney *U* test were performed to compare demographic and clinical characteristics between iRBD patients and HCs, and between iRBD patients with abnormal gait parameters and those with normal gait. Pearson’s correlation coefficient was used to analyze the relationship between gait parameters and clinical characteristics. Statistical analyses were performed with SPSS v19 software (SPSS Inc, Chicago, IL, United States). *P* < 0.05 was considered statistically significant; correlations were determined based on | r| values as follows: 0.8–1.0, very strong; 0.6–0.8, strong; 0.4–0.6, moderate; 0.2–0.4, weak; and 0.0–0.2 very weak/no correlation.

## Results

### Demographic and Clinical Characteristics of the Study Population

A total of 51 participants (31 iRBD and 20 HCs) were enrolled. There were no statistically significant differences in age, body mass index, and MoCA score between the iRBD patients and HCs; the percentage of females was lower in the iRBD group than in the HC group as expected. iRBD patients had significantly higher UPDRS III score (*U* = 471, *P* = 0.001), TOI score (T_49_ = −4.023, *P* < 0.001), NMSS (*U* = 606.5, *P* < 0.001), and RBDQ-HK score (*U* = 617.5, *P* < 0.001) compared to HCs (Table 1). The UPDRS III score of iRBD patients ranged from 0 to 5.

**TABLE 1:** Demographic and clinical characteristics of iRBD patients and healthy controls.

	iRBD (*n* = 31)	HC (*n* = 20)	*P* value
Age, years	69 (63, 73)	70 (67, 73)	0.602*
Sex, female	5 (16.1%)	11 (55%)	0.003
Height, cm	169.0 ± 7.8	162.0 ± 6.7	0.002
BMI	24.63 ± 3.35	24.68 ± 3.26	0.957
UPDRSIII	2 (0, 3)	0 (0, 0)	0.001*
MoCA	24 ± 3	25 ± 3	0.330
TOI	2.8 ± 0.5	2.2 ± 0.6	< 0.001
RBDQ-HK	39 (30, 46)	4 (3, 6.75)	< 0.001*
NMSS	37 (25, 46)	8 (2, 11)	< 0.001*

*Data are presented as mean value ± standard deviation for normally distributed data or as median (upper quartile, lower quartile) for non-normally distributed data.*

*^∗^*P* value calculated with the Mann–Whitney *U* test.*

*BMI, body mass index; F, female; HC, healthy control; iRBD, idiopathic rapid eye movement sleep behavior disorder; MoCA, Montreal Cognitive Assessment; NMSS, Non-motor Symptoms Scale; RBDQ-HK, Rapid Eye Movement Sleep Behavior Disorder Questionnaire – Hong Kong; TOI, threshold of olfactory identification; UPDRS III, Unified Parkinson’s Disease Rating Scale Part III.*

### Differences in Gait Measures Between iRBD Patients and HCs

#### Group Effect

**FIGURE 2:**
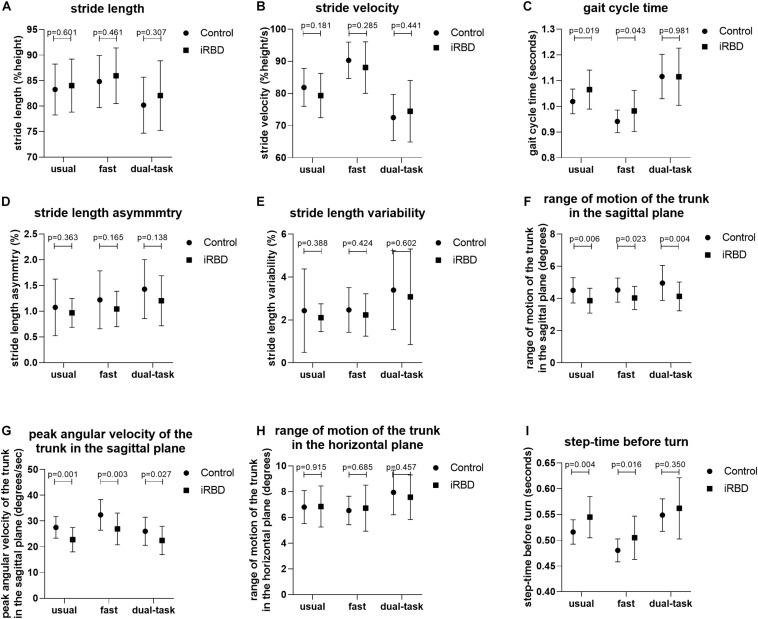
Comparison of gait measures between groups under different walking conditions. **(A–I)** Group differences in stride length **(A)**, stride velocity **(B)**, stride time **(C)**, stride length asymmetry **(D)**, stride length variability **(E)**, range of motion of the trunk in the sagittal plane **(F)**, peak angular velocity of the trunk in the sagittal plane **(G)**, range of motion of the trunk in the horizontal plane **(H)**, and step time before turn **(I)** between iRBD patients and HCs under the usual pace, fast pace, and dual-task walking conditions.

General gait measures including normalized stride length, normalized stride velocity, stride time, stride length asymmetry, stride length variability, and stride time variability did not differ between iRBD patients and HCs under usual, fast, and dual-task walking conditions (Table 2). However, iRBD patients had a significantly decreased range of motion of the trunk in the sagittal plane compared to HCs (*F*_2,152_ = 9.383, *P* = 0.004), especially in the usual and dual-task conditions (3.86 ± 0.77 vs. 4.50 ± 0.79, *P* = 0.006; 4.13 ± 0.89 vs. 4.96 ± 1.09, *P* = 0.004) (Table 3). The corresponding peak angular velocity of the trunk in the sagittal plane was also reduced in the patients (*F*_2,152_ = 11.588, *P* = 0.001). As expected, trunk motion in the horizontal plane did not differ between groups. An increase in the time for the last step before turning was observed in iRBD patients compared to controls (*F*_2,152_ = 4.724, *p* = 0.035), which was more prominent under usual and fast walking conditions. The difference between groups was also significant after adjusting for age, sex, and height (Table 3). Comparisons of gait measures between groups under different walking conditions are shown in Figure 2.

**TABLE 2:** Differences in general gait measures between iRBD patients and healthy controls.

Parameter	iRBD	HC	*P*	P*
**Normalized stride length, % height**				
Usual	84.03 ± 5.19	83.25 ± 4.99		
Fast	85.95 ± 5.45	84.82 ± 5.12		
Dual-task	82.06 ± 6.82	80.19 ± 5.49		
Group effect			0.414	0.727
Condition effect			<0.001	< 0.001
Interaction: group × condition			0.409	
**Normalized stride velocity, % height/s**				
Usual	79.33 ± 6.89	81.86 ± 5.89		
Fast	88.04 ± 8.02	90.28 ± 5.66		
Dual-task	74.44 ± 9.57	72.50 ± 7.19		
Group effect			0.617	0.614
Condition effect			<0.001	< 0.001
Interaction: group × condition			0.099	
**Stride time, s**				
Usual	1.06 ± 0.08	1.02 ± 0.05		
Fast	0.98 ± 0.08	0.94 ± 0.04		
Dual-task	1.11 ± 0.11	1.12 ± 0.09		
Group effect			0.143	0.238
Condition effect			<0.001	< 0.001
Interaction: group × condition			0.176	
**Stride length asymmetry, %**				
Usual	0.97 ± 0.28	1.07 ± 0.55		
Fast	1.04 ± 0.34	1.22 ± 0.56		
Dual-task	1.20 ± 0.49	1.43 ± 0.57		
Group effect			0.102	0.085
Condition effect			<0.001	0.004
Interaction: group × condition			0.710	
**Stride length coefficient of variation, %**				
Usual	2.11 ± 0.65	2.43 ± 1.94		
Fast	2.23 ± 1.00	2.47 ± 1.04		
Dual-task	3.08 ± 2.23	3.29 ± 1.85		
Group effect			0.352	0.711
Condition effect			0.007	0.002
Interaction: group × condition			0.973	
**Stride time coefficient of variation, %**				
Usual	2.00 ± 0.63	1.75 ± 0.71		
Fast	2.36 ± 1.55	2.36 ± 1.20		
Dual-task	3.85 ± 3.48	4.00 ± 3.33		
Group effect			0.934	0.512
Condition effect			0.001	< 0.001
Interaction: group × condition			0.798	

*Data are presented as mean value ± standard deviation.*

*^∗^*P* values for stride length (% height) and velocity (% height/s) were adjusted for age and sex; the other parameters were adjusted for age, sex, and height.*

*HC, healthy control; iRBD, idiopathic rapid eye movement sleep behavior disorder.*

**TABLE 3:** Differences in gait measures related to trunk motion and turning initiation between iRBD patients and healthy controls.

Parameter	iRBD	HC	*P*	P*
**Range of motion of the trunk in the sagittal plane, °**				
Usual	3.86 ± 0.77	4.50 ± 0.79	0.006	0.113
Fast	4.03 ± 0.72	4.52 ± 0.75	0.023	0.254
Dual-task	4.13 ± 0.89	4.96 ± 1.09	0.004	0.097
Group effect			0.004	0.009
Condition effect			<0.001	0.106
Interaction: group × condition			0.409	
**Peak angular velocity of trunk in the sagittal plane, °/s**				
Usual	22.75 ± 4.71	27.53 ± 4.19	0.001	0.007
Fast	26.93 ± 6.18	32.38 ± 5.96	0.003	0.027
Dual-task	22.45 ± 5.50	26.04 ± 5.45	0.027	0.246
Group effect			0.001	< 0.001
Condition effect			<0.001	< 0.001
Interaction: group × condition			0.099	
**Range of motion of the trunk in the horizontal plane, °**				
Usual	6.85 ± 1.59	6.80 ± 1.29		
Fast	6.72 ± 1.79	6.54 ± 1.10		
Dual-task	7.57 ± 1.73	7.94 ± 1.74		
Group effect			0.912	0.287
Condition effect			<0.001	0.002
Interaction: group × condition			0.176	
**Peak angular velocity of trunk in the horizontal plane, °/s**				
Usual	21.17 ± 5.52	21.86 ± 3.87		
Fast	24.35 ± 6.23	24.91 ± 4.33		
Dual-task	23.64 ± 6.06	24.59 ± 4.70		
Group effect			0.605	0.509
Condition effect			<0.001	0.009
Interaction: group × condition			0.710	
**Step time before turn, s**				
Usual	0.54 ± 0.04	0.51 ± 0.02	0.005	0.033
Fast	0.50 ± 0.04	0.48 ± 0.02	0.018	0.090
Dual-task	0.56 ± 0.06	0.55 ± 0.03	0.445	0.768
Group effect			0.035	0.034
Condition effect			<0.001	< 0.001
Interaction: group × condition			0.973	

*Data are presented as mean value ± standard deviation.*

*^∗^*P* values were adjusted for age, sex, and height.*

*HC, healthy control; iRBD, idiopathic rapid eye movement sleep behavior disorder.*

#### Walking Condition Effect

There was significant walking condition effect on all general gait parameters examined in this study including stride length, stride velocity, stride time, stride length asymmetry, stride length variability, and stride time variability. Both iRBD patients and HCs walked more rapidly as instructed in the fast condition and slowed down in the dual-task condition (*P* < 0.001; Table 2). However, stride length asymmetry and variability of stride length and time increased under both conditions compared to the baseline (i.e., usual walking condition) in both groups, and the time of the last step before turning was increased in the dual-task test (*P* < 0.001). There was no interaction between group and walking condition for any parameter.

### Comparisons of iRBD Patients With or Without Gait Abnormality

In order to determine whether iRBD patients with greater gait abnormality were at higher risk of conversion to synucleinopathy, the iRBD group was divided into patients with and those without gait abnormality based on the mean value of peak angular velocity of the trunk in the sagittal plane. Patients with gait abnormality had a higher NMSS score and higher number of non-motor symptoms as well as a longer RBD duration compared to patients with normal gait, but the differences were not statistically significant. UPDRS III score, MoCA, TOI, and RBDQ-HK score did not differ between the 2 groups (Table 4).

**TABLE 4:** Differences in demographic and clinical characteristics between iRBD patients with and those without gait abnormality.

	Normal gait (*n* = 14)	Abnormal gait (*n* = 17)	*P* value
Age, years	67 ± 6	70 ± 6	0.188
Sex, female	4 (28.6%)	1 (5.9%)	0.087
UPDRSIII	2 (0.75, 3)	2 (0, 3)	0.922*
MoCA	24 ± 3	24 ± 3	0.784
RBD duration	8 (4.75, 9.25)	8.5 (2, 12)	0.710*
TOI	2.9 ± 0.4	2.8 ± 0.5	0.411
RBDQ-HK	42 (29.25, 44.5)	38 (30, 49)	0.570*
NMSS	36.5 (27.5, 47)	38 (24, 55.5)	0.769*
Number of non-motor symptoms	10 ± 4	11 ± 5	0.586

Data are presented as mean value ± standard deviation for normally distributed data or as median (upper quartile, lower quartile) for non-normally distributed data.

*P value calculated with the Mann–Whitney *U* test.

*F, female; HC, healthy control; iRBD, idiopathic rapid eye movement sleep behavior disorder; MoCA, Montreal Cognitive Assessment; NMSS, Non-motor Symptoms Scale; RBDQ-HK, Rapid Eye Movement Sleep Behavior Disorder Questionnaire – Hong Kong; TOI, threshold of olfactory identification; UPDRS III, Unified Parkinson’s Disease Rating Scale Part III.*

### Correlations Between Gait Parameters and Clinical Characteristics of iRBD Patients

Negative correlations were observed between age and normalized stride length (*r* = −0.555, *P* = 0.001) and between age and peak angular velocity of the trunk in the sagittal plane (*r* = −0.386, *P* = 0.032; Supplementary Table 1). Stride length asymmetry showed a moderate negative correlation with MoCA score (*r* = −0.412, *P* = 0.021) and a weak positive correlation with RBDQ-HK score (*r* = 0.368, *P* = 0.041) (Supplementary Table 1).

## Discussion

In this cross-sectional study, we investigated subclinical changes in gait characteristics in PSG-confirmed iRBD patients compared to HCs using wearable motor sensors. While there were no differences in stride length, stride velocity, stride time, stride length asymmetry, and stride length and stride time variability in the 3 walking conditions, iRBD patients showed a significantly decreased range of motion and peak angular velocity of the trunk and had a longer step time before turning than HCs. These differences were significant even after adjusting for age, sex, and height. Because of the type of sensor that was used, we did not examine step width variability in this study. There were no differences in NMSS score, number of non-motor symptoms, UPDRS III score, MoCA, TOI, RBDQ-HK score, or RBD duration between iRBD patients with and those without abnormal gait.

Previous findings on quantitative gait characteristics in iRBD patients have been inconsistent. One study found a lack of difference in step length and velocity between PSG-proven iRBD patients and HCs but when walking at a fast pace, iRBD patients showed increased gait asymmetry as well as an increase of step width variability in the dual-task walking condition (Ehgoetz Martens et al., 2019). On the contrary, decreases in velocity and cadence and an increase in stride time variability were reported in patients with probable RBD who were diagnosed with the Mayo Sleep Questionnaire (McDade et al., 2013). Meanwhile, a real-world gait monitoring study of PSG-proven iRBD patients found decreases in gait velocity, variability, and rhyme (Del Din et al., 2020). These results are at odds with our data. The inconsistency in primary gait parameters across studies may be attributable to the different gait detection methods that were used and the heterogeneity of the disease stage among RBD patients, and suggests that these parameters lack specificity and sensitivity for predicting phenoconversion to PD in iRBD patients.

In contrast to the lack of difference in general gait parameters, we observed an increase in step time before turning in iRBD patients, suggesting that they need a longer time to prepare for a change in walking direction. Moreover, this could indicate a slower initiation of gait or FOG, which is characterized by difficulty in step initiation and turning. A previous study of gait initiation in RBD patients found that during the propulsive phase, a posterior shift in the center of pressure occurred only in iRBD patients and PD patients with FOG and not in controls or PD patients without FOG (Alibiglou et al., 2016). Thus, it is possible that some iRBD patients develop difficulty in initiating turning prior to the emergence of full motor symptoms. In fact, the pathophysiologic mechanisms of RBD and gait disturbance both involve upper brainstem structures such as the pedunculopontine nucleus (PPN) (Steriade, 2004). Activity in the PPN increases during REM sleep, which plays an important role in turning on REM sleep and maintaining atonia during this sleep stage (Rye, 1997). Moreover, the PPN is one of the nuclei that is affected by alpha-synuclein aggregation in the prodromal stage of PD, which is consistent with the elevated risk of parkinsonism associated with iRBD. Deep brain stimulation of PPN was shown to improve gait disturbance (including FOG) in PD patients (Ferraye et al., 2010; Moro et al., 2010; Thevathasan et al., 2018), and a functional magnetic resonance imaging study using gait imagery demonstrated that PD patients with FOG had higher activity in the mesencephalic locomotor region (Snijders et al., 2011), which comprises the PPN and midbrain extrapyramidal area (Alam et al., 2011). Neurotransmitter systems may also provide a link between iRBD and FOG. Gait disturbance is generally dopamine-resistant and animal experiments have indicated that it is more closely related to the cholinergic system (Karachi et al., 2010), which was found to be dysregulated in patients with PD or iRBD and associated with RBD symptoms (Müller and Bohnen, 2013). Whether the increase in step time before turning observed in iRBD patients in our study was caused by the degeneration of cholinergic neurons that occurs in the prodromal stage of PD remains to be determined; however, our finding that iRBD patients had impaired olfactory identification compared to HCs supports a mechanistic link, as degeneration of cholinergic neurons in the basal forebrain was shown to be associated with olfactory dysfunction (Doty, 2017).

Another finding of our study is that both the range of motion and peak angular velocity of the trunk in the sagittal plane were decreased in iRBD patients, indicating that patients’ trunk was more rigid and inflexible compared to that of HC subjects while walking. Several studies have reported that PD patients with RBD have more prominent axial symptoms and a postural instability and gait dysfunction (PIGD) phenotype. A large community-based longitudinal study found that PD patients with probable RBD tended to have higher axial UPDRS III subscores (Duarte Folle et al., 2019). Moreover, motor symptoms deteriorated more rapidly in patients with RBD with the PIGD phenotype (Duarte Folle et al., 2019). A cross-sectional study showed that the prevalence of PIGD was higher in PD patients who reported having past or present RBD symptoms (Bugalho et al., 2011), and a cluster study of PD subtypes based on non-motor symptoms found that patients with the highest incidence (92%) of RBD symptoms exhibited the most severe gait disturbance and had the highest rate of FOG and falls (Fereshtehnejad et al., 2015). Our study provides additional evidence that iRBD patients have increased rigidity and gait disorder (i.e., the PIGD phenotype) even at the very early stage of disease.

The results of this study demonstrate the effectiveness of wearable sensors for the early quantitative detection of gait abnormality in iRBD patients, which can potentially reveal prodromal symptoms of PD and predict the time to conversion owing to the objective and sensitive nature of quantitative gait measurements (Del Din et al., 2019). Prodromal PD symptoms usually occur together because of the clustered anatomic location of brainstem structures; thus, gait abnormality in iRBD patients may reflect a more advanced disease stage and may be accompanied by additional non-motor symptoms. iRBD patients with a longer disease duration or who are progressing to synucleinopathy may be more likely to demonstrate gait abnormality. In this study we did not observe differences in NMSS score, number of non-motor symptoms, or RBD duration between iRBD patients with and those without gait abnormality, possibly because of the small sample size and variable time course of phenoconversion to PD. As expected, we found no difference in UPDRS III score between iRBD patients with vs. those without gait abnormality, as this scale is less objective and sensitive than quantitative gait measurements. We also found that gait parameters of iRBD patients were associated with older age, cognitive impairment, and the severity of non-motor and RBD symptoms, possibly reflecting the extent of neurodegeneration in this group.

Major limitations of our study were the small sample size and cross-sectional design. We are still following the iRBD cohort annually and additional studies are underway to better characterize motor symptoms and phenoconversion in the iRBD cohort.

In summary, we found that PSG-confirmed iRBD patients exhibited decreased trunk motion while walking and increased step time before turning, which may be early manifestations of body rigidity and possible FOG as prodromal symptoms of PD. Comprehensive analyses of gait and postural balance are necessary in the follow-up of our patients to monitor for potential progression to PD. Additionally, a large longitudinal study of iRBD patients is needed to determine whether the PIGD phenotype and axial symptoms persist after conversion to PD.

## Data Availability Statement

The raw data supporting the conclusions of this article will be made available by the authors, without undue reservation.

## Ethics Statement

The studies involving human participants were reviewed and approved by Ethics Committee of Xuanwu Hospital Capital Medical. The patients/participants provided their written informed consent to participate in this study.

## Author Contributions

S-YL and PC designed the study. S-SC, YL, HZ, Z-QG, WM, J-HM, Y-TZ, and E-HX collected the data. CH provided guidance on statistical methods. LM and S-YL performed statistical analyses and drafted the manuscript. PC reviewed and critiqued the manuscript. All authors read and approved the final version of the manuscript for publication.

## Conflict of Interest

The authors declare that the research was conducted in the absence of any commercial or financial relationships that could be construed as a potential conflict of interest.
