# Supplementary material for: Detection of Motor Dysfunction With Wearable Sensors in Patients With Idiopathic Rapid Eye Movement Disorder
Source: Front Bioeng Biotechnol. 2021 Apr 15;9:627481. doi: 10.3389/fbioe.2021.627481 (PMC8084288; doi:10.3389/fbioe.2021.627481)
Supplement: Supplementary file 1 [file Table_1.DOCX]

# Supplementary Table 1 Correlations between gait parameters and clinical characteristics in iRBD patients

|  | age | | UPDRS III | | RBD duration | | MoCA | | TOI | | NMSS | | RBDQ-HK | |
| --- | --- | --- | --- | --- | --- | --- | --- | --- | --- | --- | --- | --- | --- | --- |
|  | r | P | r | P | r | P | r | P | r | P | r | P | r | P |
| Normalized stride length | -0.555 | 0.001* | -0.208 | 0.262 | -0.055 | 0.768 | 0.275 | 0.134 | -0.188 | 0.310 | -0.358 | 0.048* | -0.175 | 0.346 |
| Normalized stride velocity | -0.248 | 0.179 | 0.030 | 0.875 | -0.023 | 0.900 | 0.043 | 0.817 | 0.035 | 0.853 | -0.142 | 0.445 | -0.242 | 0.191 |
| Gait cycle time | -0.143 | 0.443 | -0.233 | 0.207 | 0.000 | 1.000 | 0.169 | 0.365 | -0.238 | 0.197 | -0.123 | 0.510 | 0.153 | 0.412 |
| Range of motion of the trunk in the sagittal plane | -0.338 | 0.063 | -0.003 | 0.989 | 0.073 | 0.698 | 0.023 | 0.904 | -0.001 | 0.995 | -0.221 | 0.231 | -0.169 | 0.363 |
| Peak angular velocity of the trunk in the sagittal plane | -0.386 | 0.032* | 0.039 | 0.836 | 0.098 | 0.600 | 0.215 | 0.246 | 0.093 | 0.619 | -0.148 | 0.428 | -0.053 | 0.778 |
| Range of motion of the trunk in the horizontal plane | -0.261 | 0.156 | -0.149 | 0.424 | -0.047 | 0.801 | 0.263 | 0.152 | -0.070 | 0.706 | -0.094 | 0.616 | -0.142 | 0.447 |
| Peak angular velocity of the trunk in the horizontal plane | -0.059 | 0.754 | -0.049 | 0.794 | 0.130 | 0.487 | 0.104 | 0.577 | -0.063 | 0.736 | 0.236 | 0.202 | -0.028 | 0.882 |
| Step-time before turn | -0.145 | 0.437 | -0.246 | 0.183 | 0.081 | 0.664 | 0.135 | 0.471 | -0.219 | 0.237 | -0.167 | 0.368 | 0.136 | 0.466 |
| Stride length asymmetry | 0.293 | 0.110 | 0.110 | 0.556 | 0.010 | 0.959 | -0.412 | 0.021* | 0.101 | 0.587 | 0.326 | 0.074 | 0.368 | 0.041* |
| Stride length coefficient of variation | 0.101 | 0.589 | -0.058 | 0.756 | -0.304 | 0.096 | -0.265 | 0.149 | -0.174 | 0.351 | 0.184 | 0.323 | 0.244 | 0.185 |
| Stride time coefficient of variation | 0.052 | 0.781 | -0.231 | 0.212 | 0.065 | 0.727 | 0.197 | 0.287 | -0.073 | 0.695 | 0.096 | 0.608 | 0.242 | 0.189 |

Abbreviation: iRBD, idiopathic rapid eye movement sleep behavior disorder; UPDRS III, the third part of Unified Parkinson’s Disease Rating Scale; MoCA, Montreal Cognitive Assessment; TOI, the threshold of olfactory identification; RBDQ-HK, Rapid-eye-movement Sleep Behavior Disorder Questionnaire HongKong; NMSS, Non-Motor Symptoms Scale.
